# A Cross-Sectional Study of Drowning in Saudi Arabia Using First Responder Data

**DOI:** 10.1038/s41598-025-04317-5

**Published:** 2025-07-01

**Authors:** Kholood K. Altassan, Reema M. Alhussein, Rawan T. Ghandour, Khalid A. Aldossari, Naif A. Aldossari, Meshal K. Alghamdi, Fahad Almutlaq, Yousef Mohammad Alsofayan, Mohammed K. Alageel

**Affiliations:** 1 Department of Family and Community Medicine, College of Medicine, King Saud University, Riyadh, Saudi Arabia; 2 Department of Emergency Medicine, Prince Sultan Military Medical City, Riyadh, Saudi Arabia; 3 Department of Emergency Medicine, College of Medicine, King Saud University, Riyadh, Saudi Arabia; 4 College of Medicine, King Saud University, Riyadh, Saudi Arabia; 5 Department of Geography, College of Humanities and Social Sciences, King Saud University, Riyadh, Saudi Arabia; 6 Medical Affairs Department, Saudi Red Crescent Authority, Riyadh, Saudi Arabia; 7 Department of Emergency Medicine, University of British Columbia, Vancouver, Canada

**Keywords:** Epidemiology, Risk factors

## Abstract

Drowning is a significant public health issue and is considered one of the most avoidable yet underappreciated causes of mortality worldwide. Drowning likely presents a significant public health burden in Saudi Arabia, especially in children. To our knowledge only three studies have been conducted investigating drowning in Saudi Arabia, all of which were single center hospital-based studies. This is the first national cross-sectional study to assess drowning epidemiology in Saudi Arabia and the first to utilize first responder data. This study’s main objectives are to quantify the burden of drowning in Saudi Arabia, and describe the epidemiological profile and geographic distribution of drowning cases across the country. We conducted a cross-sectional study using electronic data from the Saudi Red Crescent Authority (SRCA) on drowning incidents between January 2019 to November 2021. Descriptive statistics such as frequencies and percentages were calculated using the Statistical Package for the Social Sciences (SPSS), software for Windows (version 23.0). Men and children under 4 had the highest incidence of drowning, with 2021 incidence rates of 19.86 and 54.08 per million, respectively. The regions of Makkah, Riyadh, and Dammam, had the largest number of cases, accounting for 30.49% (*n* = 501), 19.84% (*n* = 326), and 13.02% (*n* = 214) of all cases respectively. The regions with the highest drowning incidence in 2021 were Tabuk, Al-baha, Makkah, Jizan, and Hail with incidence rates of 32.32, 27. 96, 22.28, 20.97, and 20.5 per million people, respectively. Drowning is a leading cause of death in Saudi Arabia and globally, with young children most at risk. Our study identifies data gaps and high-incidence areas requiring further investigation. The low bystander cardiopulmonary resuscitation (CPR) rate and high mortality call for better public education on drowning prevention and first aid.

## Introduction

Drowning is a significant but often under recognized public health issue, despite being largely preventable. In spite of increasing incidence globally, preventive measures do not receive adequate attention^1^. In Saudi Arabia there is growing interest in addressing drowning prevention although local data is sparse^2^.

In 2019, an estimated 236,000 people died from drowning. It was the 3rd leading cause of unintentional injury-related deaths, accounting for 7% of all cases^3^. Many studies have suggested that figures representing the burden of drowning are likely underestimating the true burden as they often exclude water transport and disaster-related drowning^4^. Additionally, the majority of all unintentional drownings, approximately 92%, are attributed to low and middle income countries (LMIC) where there are limited resources allocated to data collection^3^.

Despite the overall paucity of available data, some studies have provided insight into the financial burden of drowning. In the United States (US) the annual cost of drowning is $273 million US dollars annually, while the overall yearly cost of drowning injuries in Australia and Canada is estimated at $85.5 million and $173 million, respectively^5^. Globally, the annual cost of drowning mortality is estimated at $146.9 billion based on 2015 data. Moreover, the cost of drowning is disproportionately large in many low-income countries and can amount to more than 0.8% of the GDP^6,7^.

Drowning has several clearly identifiable risk factors with age being the most consistent. Multiple studies have shown infants and young children from 1 to 4 years old account for the majority of drowning cases^8,9^. Factors that may contribute to drowning mortality include lack of, or overestimation of, swimming ability, propensity for risky behavior, developmental disorders, hypothermia, and myocardial infarctions^10^. Environmental factors such as the presence of physical barriers between people and water, covered or protected water supplies, safe water crossings, as well as the availability of public health regulation and community education can also influence drowning risk^11^. There is a wide disparity in the burden of drowning relative to a country’s GDP, with the incidence of drowning in (LMICs) being more than three times that of high-income countries (HICs). Additionally, most drowning in (LMICs) occurs in natural waters, such as ponds, seas, and wells, whereas drowning in private or public pools is more common in (HICs)^8^.

A recent systematic review of drowning in the Eastern Mediterranean region demonstrated that fatal drowning rates varied significantly from 0.48 to 18.5 deaths per 100,000. The study also identified risk factors such as male gender, age between 1 and 4, and residential status^12^. A study investigating pediatric drowning cases in Oman from 2008 to 2017 found that two-thirds of the cases were under the age of 6, a swimming pool was the scene of nearly 60% of incidents, and almost a third of children were unsupervised during the incident. Additionally the study reported that more than half the victims required cardiopulmonary resuscitation (CPR)^13^. A paper from Jordan exploring variables that were associated with local drowning mortality, also reported male gender and young age as likely factors. They further showed that most cases occurred in the month of August, were accidental in nature, and drugs and alcohol were rarely identified in the victims^14^.

In Saudi Arabia, few papers have been published addressing drowning, all of which are single center studies in pediatric patients who had survived transport to hospital^15–18^. None of the studies have addressed the community burden, predictive risk factors or relevant outcomes of drowning in the local population. The country’s demographic and social make-up as well as its geographical location between two seas lends it to having a high burden of drowning. Population-based studies are a necessary first step for designing targeted interventions and preventive measures.

The main objectives of this study are to quantify the burden of drowning in Saudi Arabia, and to describe the epidemiological profile and geographic distribution of drowning cases across the country. This is the first national-level investigation of drowning in Saudi Arabia and the first to utilize data from first responder services. This study will provide a baseline of the local burden of drowning, identify trends in incidence, and regional clusters, and help to highlight modifiable risk factors that could be the focus of future public health interventions.

## Methodology

We conducted a cross-sectional study using electronic data from the Saudi Red Crescent Authority (SRCA), which acts as the primary national pre-hospital emergency medical service (EMS) responding to all community medical emergency incidents in Saudi Arabia^19^. The studied time period was between January 2019 to November 2021. Variables reported in the dataset included patient demographics (age, gender, nationality), region, time of reporting to EMS, incident location, mode of communication with EMS, time to arrival of EMS, whether CPR was performed by the EMS team, and the patient’s status upon arrival of EMS. Incident location was reported via geotagging. Based on the Ministry of Interior’s classification, locations were categorized as either:^1^ ‘in city’—within urban boundaries, or^2^ ‘out city’—referring to incidents in rural areas, highways, or agricultural roads. Mode of communication with EMS included phone services either 911 or 997, and use of mobile applications able to provide EMS geolocated response “Tawakkalna” or “Asafny”.

The study was reviewed and approved by the Institutional Review Board (IRB) at the SRCA on 28/2/2022, IRB log number: 22-08E. The need for informed consent was waived by the IRB. All study methods were performed in accordance with the relevant guidelines and regulations. The statistical analysis was performed using the Statistical Package for the Social Sciences (SPSS), software for Windows (version 23.0). Descriptive statistics such as frequencies and percentages were calculated.

## Results

**Fig. 1:**
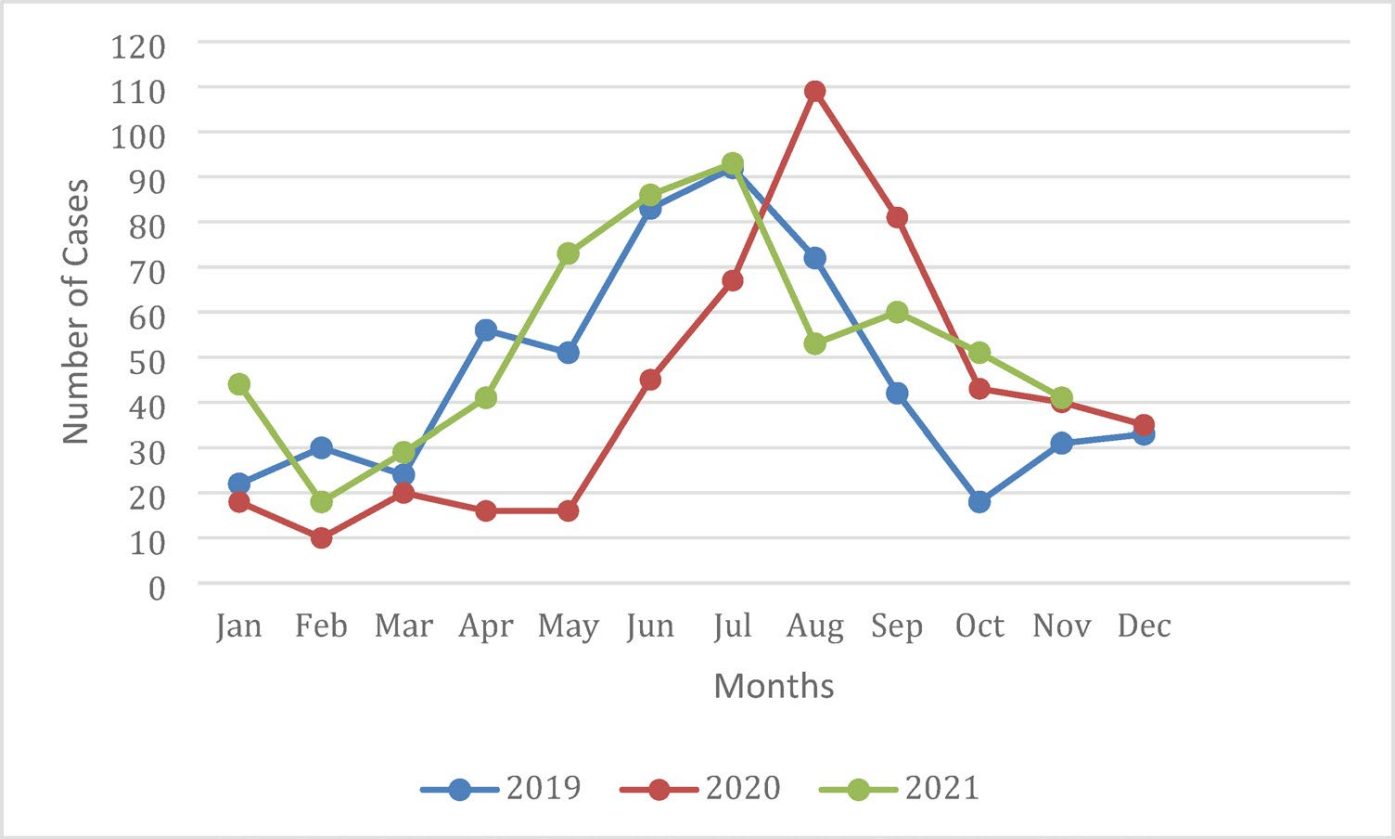
Seasonal distribution of drowning incidents in Saudi Arabia (2019–2021).

This study evaluated a total of 1643 drowning cases across all regions of Saudi Arabia collected through the SRCA database from January 2019 to November 2021. Overall, the highest numbers of incidents were reported in the summer, between May and September, with only slight variations between years. (Fig. 1) The annual national incidence of drowning in our dataset ranged from 15.8 to 18.4 per million and varied by region and demographic groups represented in (Tables 1 and 2).

**Table 1:** Drowning incidents in Saudi Arabia per 1,000,000 people for each region, from January 2019 to November 2021.

Region	*N* (total study period)	% of total cases	Number of cases 2019	Number of cases 2020	Number of cases 2021	Cases per million 2019*	Cases per million 2020*	Cases per million 2021*
Arar	14	0.85	2	6	6	5.8	16.6	15.30
Asir	122	7.43	42	48	32	22.4	24.5	15.09
Al-Baha	21	1.28	3	8	10	9.4	24.1	27.96
Ad-Dammam	214	13.02	70	65	79	14.8	13.0	14.84
Hail	40	2.43	16	8	16	23.2	11.1	20.50
Jizan	93	5.66	37	25	31	28.1	18.2	20.97
Aljouf	20	1.22	4	10	6	7.2	17.3	9.57
Al-Madinah	111	6.76	36	36	39	18.0	17.2	17.41
Makkah	501	30.49	175	139	187	23.0	17.4	22.28
Najran	17	1.03	3	6	8	5.5	10.4	12.92
Al-Qassim	75	4.56	25	29	21	19.8	22.1	14.93
Al-Riyadh	326	19.84	108	94	124	13.5	11.2	13.90
Tabuk	89	5.42	33	26	30	40.2	30.4	32.32
Total	1643	100	554	500	589	18.4	15.8	17.54

* Based on population census data from the Saudi General Authority for Statistics (GSTAT)^20^.

**Table 2:** Drowning incidents in Saudi Arabia per 1,000,000 people by age, sex, and nationality, from January 2019 to November 2021.

	Number of cases	% of total cases	Number of cases 2019	Number of cases 2020	Number of cases 2021	Cases per million 2019**	Cases per million 2020**	Cases per million 2021**
Age group								
0–4	294	28.97	45	92	157	16.03	33.09	54.08
5–9	134	13.20	9	45	80	3.39	16.55	26.95
10–14	67	6.60	6	18	43	2.54	7.34	15.83
15–19	64	6.31	10	20	34	4.84	9.48	14.51
20–24	74	7.29	6	25	43	2.62	10.05	16.53
25–29	107	10.54	8	44	55	2.41	12.35	14.91
30–34	68	6.70	7	30	31	1.86	7.55	7.56
35–39	62	6.11	7	28	27	2.16	8.28	7.50
40–44	41	4.04	4	20	17	1.73	7.95	6.35
45–49	24	2.36	2	7	15	1.15	3.87	7.86
50–54	27	2.66	2	11	14	1.50	7.79	9.48
55–59	18	1.77	1	5	12	1.12	5.18	11.50
60–64	13	1.28	1	7	5	1.81	11.55	7.51
65+	22	2.17	3	9	10	4.07	11.48	11.29
Nationality								
Saudi	673	66.31	74	222	377	4.23	12.35	18.80
Non-Saudi	342	33.69	37	139	166	2.95	10.24	12.27
Sex								
Male	780	76.85	90	287	403	4.91	14.89	19.86
Female	235	23.15	21	74	140	1.79	6.03	10.51
Total reported	1015	61.78*	111	361	543	3.69	11.44	16.17
Total	1643		554	500	589			
% reported	61.8%		20%	72.2%	92.2%			

*This is the percentage of cases with reported demographics from all cases in the data set.

**Based on population census data from the Saudi General Authority for Statistics (GSTAT)^20^.

Demographic data were reported for 62.8% of the cases (*n* = 1,015). Among those the majority were Saudi (*n* = 673, 66.31% ) and male (*n* = 780, 76.85% ), while a smaller proportion were non-Saudi (*n* = 342, 33.69% ) or female (*n* = 235, 23.15% ). The incidence per million people ranged from 4.23 to 18.8 for Saudis compared to 2.95 to 12.27 per million non-Saudis. (Table 2) Children aged 0–4 years accounted for 29% (*n* = 294) of all drowning cases, with incidence rising from 16.03 to 54.08 per million between 2019 and 2021. The second highest was reported amongst 5 to 9 year olds accounting for 13.2% (*n* = 134) of drowning and an incidence rate up to almost 27 cases per million per year. In contrast the 30 to 54 year age groups, covering over a quarter of all cases, had the lowest incidence rates ranging from 1.15 to 9.48 per million per year (Table 2).

**Fig. 2:**
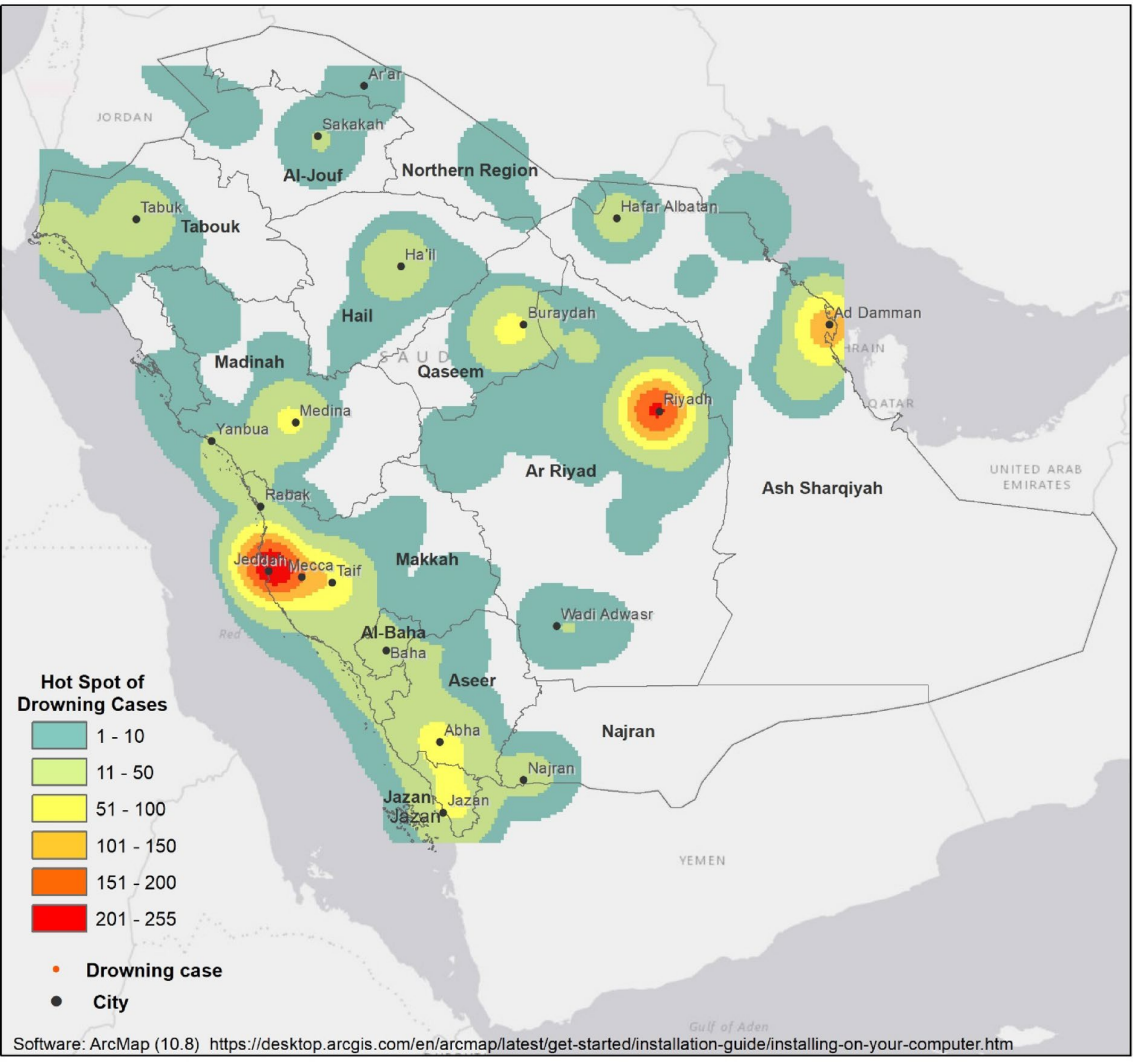
Geographical distribution of drowning incidents in Saudi Arabia (2019–2021).

Geographical distribution of drowning cases throughout the study period varied significantly between the 13 regions in Saudi Arabia. At first glance it is clear that the three most populated regions, Makkah, Riyadh, and Dammam, had the largest number of cases, accounting for 30.49% (*n* = 501), 19.84% (*n* = 326), and 13.02% (*n* = 214) of all cases, respectively. Looking at annual incidence rates we see that some of the regions representing the smallest proportion of total drowning cases have some of the highest incidence rates in the country exceeding the national incidence rate. The regions with the highest drowning rates in 2021 were Tabuk, Al-baha, Makkah, Jizan, and Hail with incidence rates of 32.32, 27. 96, 22.28, 20.97, and 20.5 per million people, respectively. In 2019 Tabuk had a drowning rate of 40.2 per million people, nearly double the incidence rate in Makkah, but accounted for only 5.42% of all cases in the study period. Geographical distribution of cases is presented in Table 1; Fig. 2.

Regarding mode of communication with EMS, the majority of cases were reported by phone (*n* = 1599, 97.3%). Digital applications were utilized in less than 3% of cases (*n* = 44). Only 8 drowning victims received CPR, almost always by an EMS responder. In about 54% of cases (*n* = 893) the victim was picked up by EMS. Among the 750 cases that were not picked up, most refused pick-up (*n* = 306, 40.8%) while almost a third (*n* = 211, 28.13%) were dead upon EMS arrival. Most drowning cases were within city limits while 539 cases were outside the city. In more than 60% cases (*n* = 1004) EMS took less than 15 min to arrive and in more than a quarter of cases they took between 15 and 30 min. In nearly 4% of cases (*n* = 60) EMS arrival took between 1 and 3 h. (Table 3)

**Table 3:** Characteristics of emergency medical services (EMS) communication and response for all drowning cases in Saudi Arabia from January 2019 to November 2021.

		*N*	%
Mode of communication with EMS	997	1352	82.29
	911	247	15.03
	Electronic paramedic	32	1.95
	Tawakkalna app	11	0.70
	Asafny app	1	0.06
CPR was performed	No	1635	99.51
	Yes	8	0.49
CPR performed by	SRCA	6	75
	A family member	1	12.50
	First responder	1	12.50
Picked up by EMS	Yes	893	54.35
	No	750	45.65
Reason for “No Pick-up” (out of 750)	Patient refused the transfer	306	40.80
	Death	211	28.13
	No injury	106	14.13
	On-site treatment provided	54	7.20
	Patient not found	48	6.40
	Transfer by another ambulance	24	3.20
	False incident	1	0.13
Within city boundaries	No	1104	67.19
	Yes	539	32.80
EMS time to arrival	15 min or less	1004	61.10
	Between 15 to 30 min	442	26.90
	Between 30 to 60 min	137	8.34
	Between 60 to 180 min	40	2.43
	More than 180 min	20	1.22

*Percentage is calculated out of the total number of cases 1643 except for “CPR performed by” and “Reason for no pick-up” where it is calculated out of 8 and 750, respectively. *CPR* Cardiopulmonary resuscitation, *SRCA* Saudi Red Crescent.

Figure 2 depicts a spatial cluster analysis illustrating high (hot spots) and low values of drowning cases in Saudi Arabia. Drowning cases have been classified into six levels based on the number of cases: (1–10), (11–50), (51–100), (101–150), (151–200), and (201–255).

## Discussion

### National burden and epidemiology of drowning

Drowning is a leading cause of morbidity and mortality globally^21^, yet its national burden in Saudi Arabia has not been previously quantified. In our dataset we found the national incidence of reported drowning ranged between 15.8 per million (2020) and 18.4 per million (2019). In 2021 the incidence was 17.54 per million. The slight variation in incidence between years is possibly related to regional fluctuations in reported cases, or due to minor changes in risk factors^5^. As our dataset did not include post-transfer mortality outcome we could not compare to global drowning mortality rates. As our dataset did not include post-transfer mortality outcome we could not compare to global drowning mortality rates. Incidence of all outcome drownings (fatal and nonfatal) is rarely published and often significantly underestimated as incidents that are resolved without medical intervention are unlikely to be reported^22^.

### Seasonality and temporal trends

The results from this study demonstrated a seasonal pattern where the highest number of incidents were reported during the warmest months of the year, May through September, when people are most likely to engage in recreational water activities. In 2020 this seasonal peak was pushed forward slightly, likely due to COVID-19 lockdown measures imposed through mid-summer^23^.

### Demographic risk factors

Global evidence, including a study from the United States (1980–1995) and a systematic review from the Middle East (2022), suggests that young age, less than 5, is a significant risk factor for drowning^12,24^. According to The Centers for Disease Control (CDC), drowning is more common among children aged 1–4 years than any other age group^25^. Our study also found that most victims of drowning were below five years of age. This is an important finding as designing prevention strategies need to be informed and tailored for specific age groups^24^. Strategies shown to reduce the risk of drowning in this age group include teaching swimming, enclosing pools with barriers, and ensuring adult supervision^12^. Published literature, including studies from the Eastern Mediterranean region and North America, further indicates that male gender, across various age groups, is a risk factor for drowning^12,26^. A population-based study in Canada, analyzing data from 2008 to 2012, found a male-to-female drowning ratio of 5 to 1^26^^12^. This is consistent with our study findings, where males accounted for more than three quarters of total cases and the risk of drowning given male sex was 1.9 times higher compared to females. This may reflect the higher activity of males participating in swimming activity locally and an increased likelihood to engage in risky behavior. Additionally we found that Saudis had a higher incidence of drowning compared to non-Saudis. This may be because most non-Saudi residents are foreign workers^20^, thus their age is higher than that of the majority of drowning cases. Further, many foreign workers are laborers with lower socio-economic status^27^, thus they are less likely to have time and access for participating in recreational swimming. Yet, this group still contributes significantly to the overall burden of drowning, which may be due to their lower socioeconomic position, insufficient swimming skills, and lack of knowledge and awareness about swimming safety.

### Geographic distribution

Geographically, drowning cases vary widely across regions in Saudi Arabia. The variation in drowning incidence between regions and the spatial distribution of clusters could be related to geography, climate, population density, socio-economic variability, local cultural and recreational behaviors, and the quality of reporting. Our analysis found that nearly two-thirds of all cases occurred in the densely populated regions of Makkah, Riyadh, and Dammam. The large number of drownings in these areas is likely reflecting their large populations. When we consider the rate of drowning; the number of drownings per million people, the top five regions with the highest drowning rates are Tabuk, Jizan, Makkah, Al-Baha, and Assir. Tabuk in particular has double the average rate of yearly drowning cases compared to other regions. This may be due to the fact that Tabuk is located on the Red Sea coast^28^, posing a risk of open-water drowning incidents, in addition to swimming pool drownings. Additionally, in the winter the region is prone to post-rain flooding due to its mountainous topography and the presence of valleys^29^. It is a common cultural activity to use these flooded areas for recreational swimming^30^. Jizan and Makkah’s high rates may also be related to both being coastal areas on the Red Sea. Makkah region also includes the major coastal city of Jeddah, which is both densely populated and a popular tourism destination, with recreational swimming activities year-round^28^. Al-Baha and Assir have the highest annual volume of rain, with topography similar to Tabuk making them also vulnerable to flooding^31^. Every year, during the rainy season, there are reports of drowning in flood waters and rain torrents in various regions of the country^30,32–34^. In contrast to the other regions we note that Hail recorded a significantly higher number of cases outside of the city boundaries. This may be related to increased activity near bodies of water surrounding the nearby valleys^35^.

### Social and environmental risk factors

The findings of this study suggest that drowning cases may be associated with both social and environmental risk factors. A 2013 review paper on drowning in Saudi Arabia observed that inadequate supervision by caretakers is an important risk factor for drowning in children under the age of 5 years^18^. A study in Aseer that looked at pediatric drowning incidents between 1999 and 2010 reported that all children that had drowned there were without adult supervision at the time^36^. Another study conducted in the eastern coastal city of Alkhobar identified poorly secured swimming pools as a significant risk factor for drowning among children^37^. Open wells and post-rain water collections in rural regions have also been mentioned as presenting a drowning risk^18^. The country’s cultural and religious norms may have resulted in limited swimming exposure, particularly amongst women, leading to lower overall swimming ability and awareness regarding drowning risks. The absence of comprehensive safety regulations, including lifeguards and warning signs in swimming areas, may also play a role. This is particularly important given that major Saudi cities lie on the coast of both the Arabian Gulf and the Red Sea, which are bodies of water with strong currents and may expose swimmers to dangerous swimming conditions.

### Prehospital CPR

Several studies have demonstrated that a favorable neurological outcome after drowning is associated with the speedy application of CPR^18,38^, particularly when provided by bystanders compared to first responders^39^. In our study, data showed only 0.5% of incidents received CPR and all but a single case were performed by a SRC paramedic or a non-SRC first responder. This finding highlights the low rate of bystander CPR which can be potentially life-saving and is likely attributed to low CPR education and training in the community.

### Study strengths and limitations

This is the first study to investigate the burden of drowning in Saudi Arabia using national EMS data. It is also the first study to calculate national incidence of drowning by age, gender, and nationality. This study also illustrates the geographical distribution of drowning cases in the country, bringing to light the significant number of coastal drownings and the unexpectedly high incidence of drowning in less populated regions.

Nevertheless, we note limitations to our study. First, we were unable to accurately estimate the national drowning mortality rate. Mortality reported in this dataset referred only to onsite death and did not include mortality after arrival of EMS teams. This likely underestimates drowning mortality in Saudi Arabia thus limiting our ability to investigate associated risk factors. Second, we suspect that the available dataset may be underappreciating the true burden of drowning as victims are often privately transported to hospital, bypassing EMS. Further, the dataset had a high rate of missing demographic data where only 60.8% of cases had associated demographic data reported. This was likely due to the utility of the electronic surveillance system being initially low when it was first launched in 2019, as evidenced by the high rate of missing data that year at nearly 80% and the subsequent decrease in missing information in 2020 (28%), and 2021 (8%). Ultimately, this led to an incomplete portrayal of drowning epidemiology in the country. Finally, the electronic dataset represents unverified cases of drowning reported to EMS, as any report with EMS dispatch is documented as a case. This also contributes to inaccurate representation of the true frequency of drowning incidents.

### Recommendations and future directions

Effective approaches to prevent drowning and reduce the associated mortality may include public education, lifeguard supervision, and applying early CPR^12^. Evidence also suggests comprehensive home safety education and free safety device programs can be beneficial^15^, as the creation of barriers around water sources on public property, which has been successful in HIC^8^. To implement drowning prevention programs effectively, timely and accurate data collection is essential for monitoring interventions and allocating public health resources^1,22^.

We recommend establishing a nationwide strategic drowning regulations framework and operational guidelines to mitigate, detect, and respond to drowning incidents in Saudi Arabia. Additionally, community engagement through awareness campaigns and the promotion of bystander CPR training is crucial, as low bystander CPR rates are a significant barrier to survival. It is also important to predict high risk areas of flooding to anticipate and plan for the deployment of specialized emergency personnel and resources.

As this study highlighted gaps in the SRCA’s electronic reporting system, we recommend enhancing data collection at the scene, along with the inclusion of prospective data points, to better identify drowning risk factors and more accurately assess morbidity and mortality. Finally, sharing epidemiological profiles and clinical characteristics with the international community and establishing drowning registries could guide decision makers in implementing long term interventions in various locations.

## Conclusion

Drowning remains a leading cause of morbidity and mortality both globally and in Saudi Arabia. This study found that the demographic distribution of drowning cases in Saudi Arabia mirrors global trends, with young children being the most affected group. Analysis of the SRCA surveillance data revealed geographic areas with higher incidence rates, highlighting the need for targeted investigation of risk factors and strategic deployment of EMS resources. The findings also point to critical gaps in data completeness and pre-hospital response, particularly the low rate of bystander CPR. These insights underscore the importance of improving surveillance systems and expanding public education on drowning prevention and first aid.

## Data Availability

The first responder data utilized in this study was provided by the Saudi Red Crescent Authority (SRCA) and can be made available to researchers upon submission of a formal request to the SRCA at nacc@srca.or.sa. All other national data can be found at the Saudi General Authority for Statistics website.
